# Supplementary material for: Bionic artificial skin with a fully implantable wireless tactile sensory system for wound healing and restoring skin tactile function
Source: Nat Commun. 2024 Jan 2;15:10. doi: 10.1038/s41467-023-44064-7 (PMC10762199; doi:10.1038/s41467-023-44064-7)
Supplement: Supplementary file 1 — Supplementary Information [file 41467_2023_44064_MOESM1_ESM.pdf]

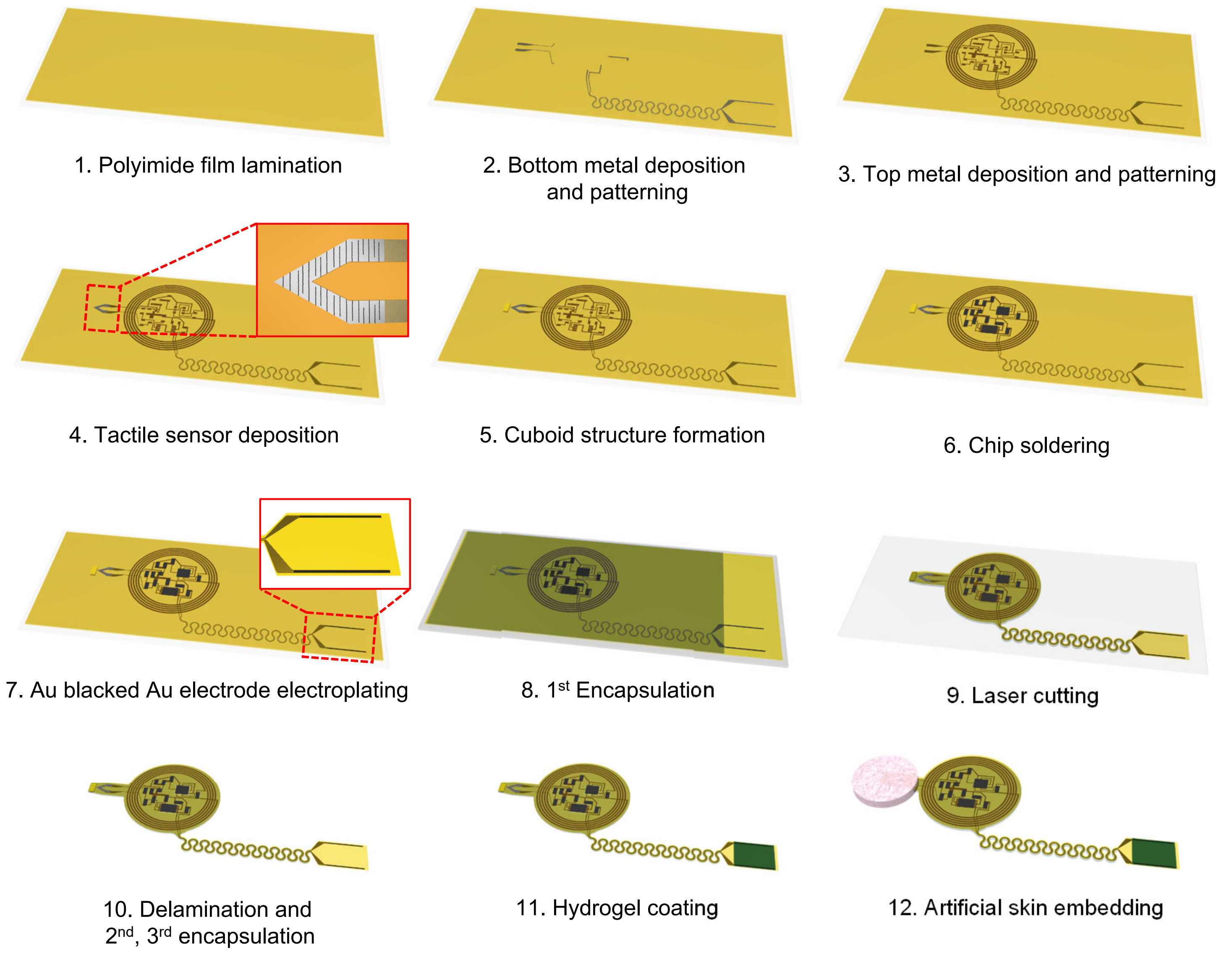

Supplementary Fig. 1 | Schematic illustration of the WTSA fabrication process. The detailed material information and fabrication conditions are described in the Methods section.

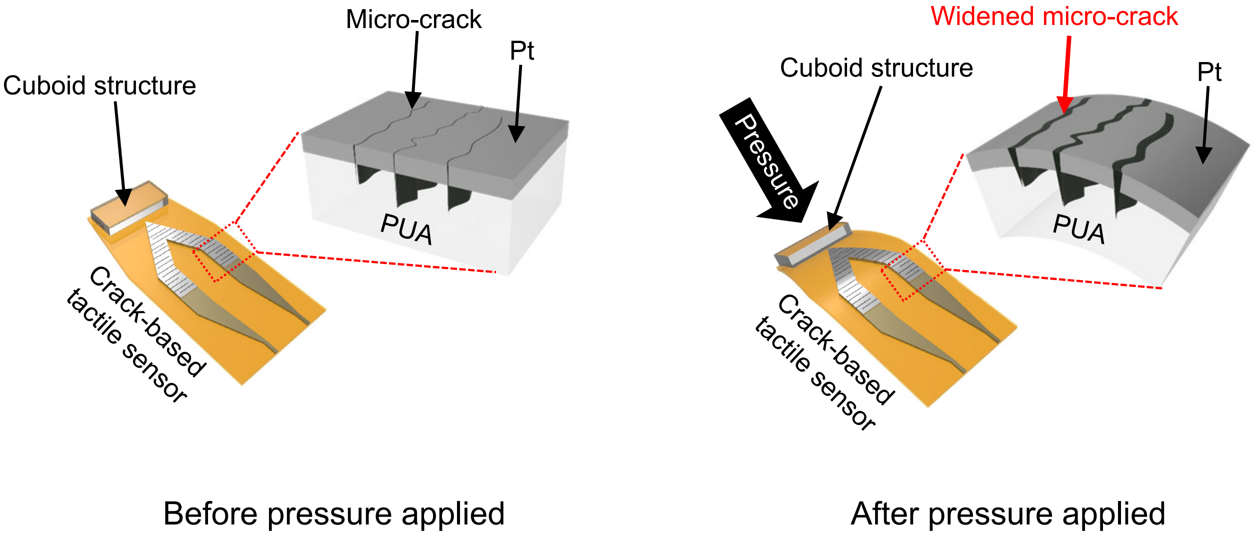

Supplementary Fig. 2 | Schematic image of the crack-based tactile sensor before and after external pressure is applied. When external pressure is applied, the cuboid structure concentrates the pressure to crack-based tactile sensor, inducing the structural deformation of micro-cracks on the Pt metal layer.

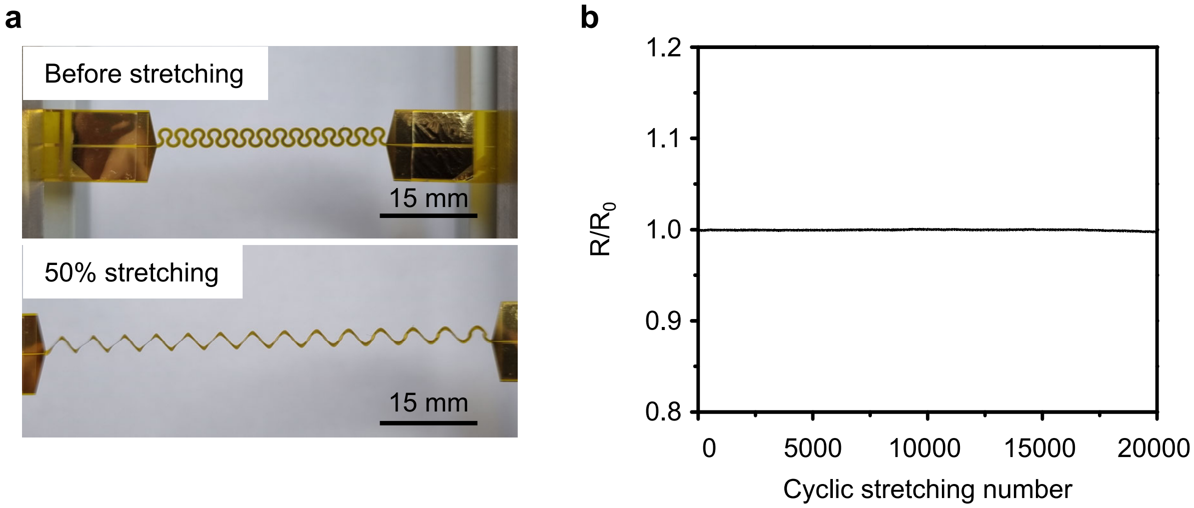

Supplementary Fig. 3 | a, Cyclic stretching test for serpentine structured interconnection between WPPFM and neural interface electrodes. **b,** Almost no resistance shift (0.42%) occurred after 20,000 times of 50% stretching test.

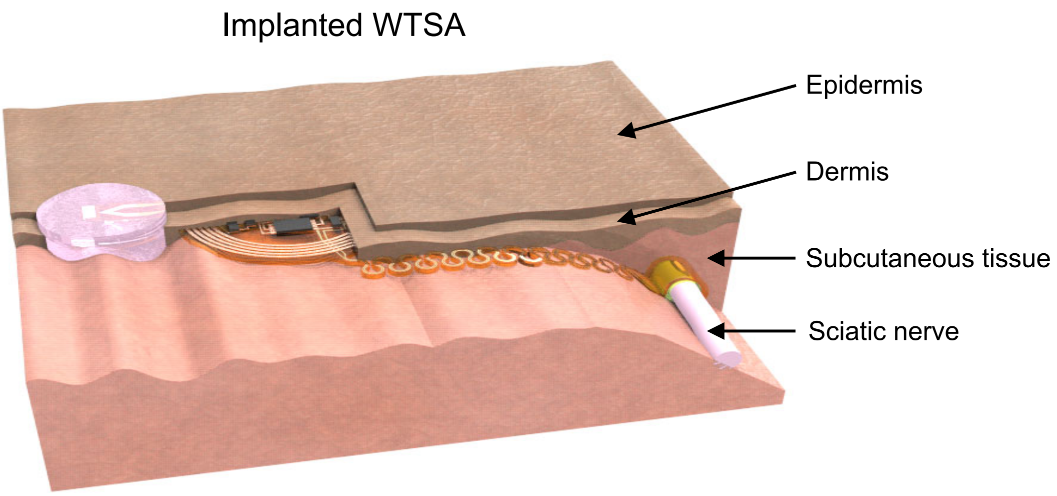

Supplementary Fig. 4 | Schematic image of the WTSA implanted on a severely damaged skin. The WPPFM of WTSA is placed in the subcutaneous tissue while hydrogel coated neural interface is placed on the sciatic nerve.

a

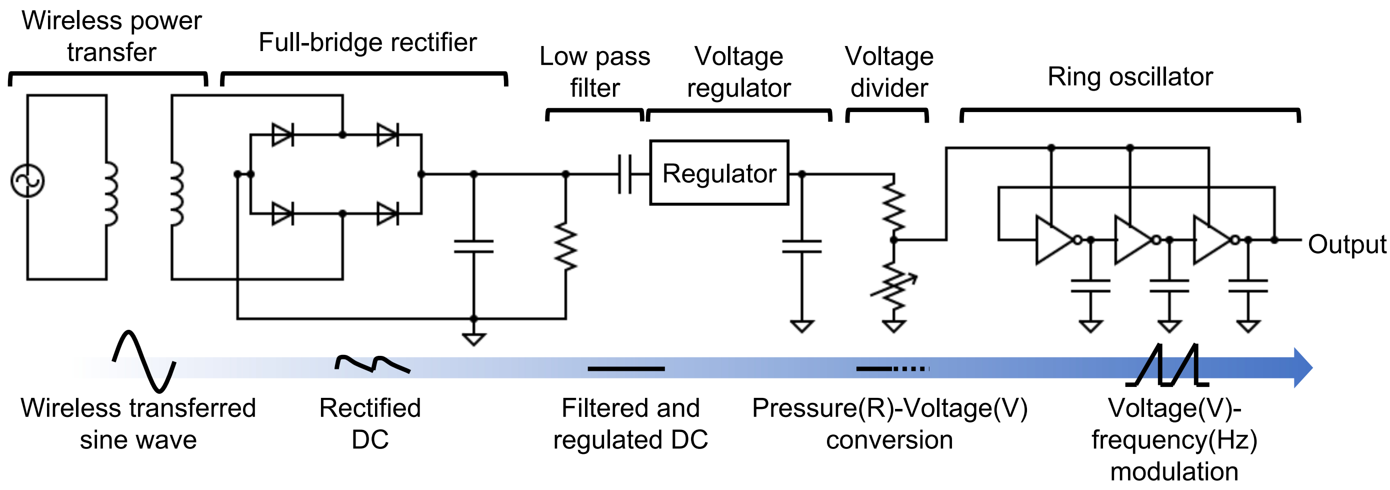

b

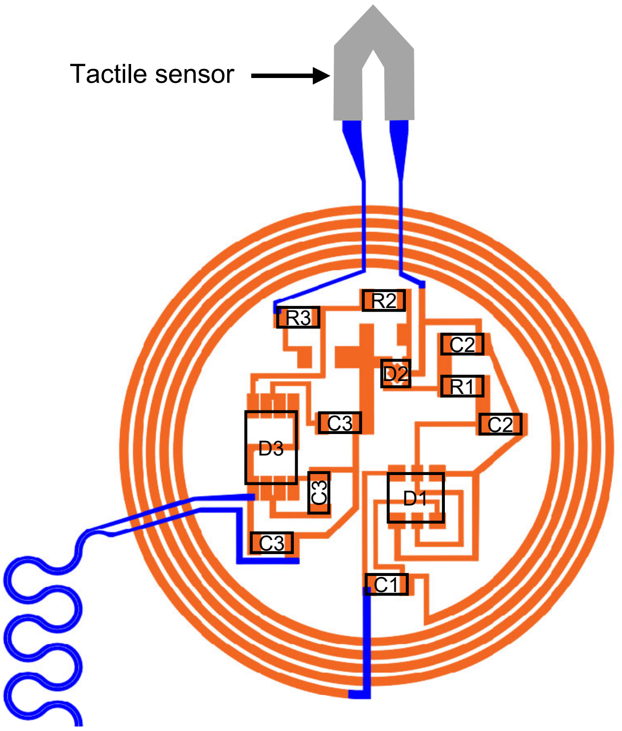

c

	Component	Product number
C1	39 pF	C0603C390J5GACTU
C2	4.7 μF	CL10A475KP8NNND
C3	1 μF	0603YC105JAT2A
R1	100 Ω	CRCW0603100RFKEAC
R2	150 Ω	RT0603DRE07150RL
R3	0 Ω	MCT06030Z0000ZP500
D1	Full-bridge rectifier	BAS40XY,115
D2	Voltage regulator	NCP110AMX120TBG
D3	Triple inverter	SN74LVC3G04DCUR

Supplementary Fig. 5 | Circuit diagram (**a**) and layout (**b**) of WPPFM with specific chip information (c).

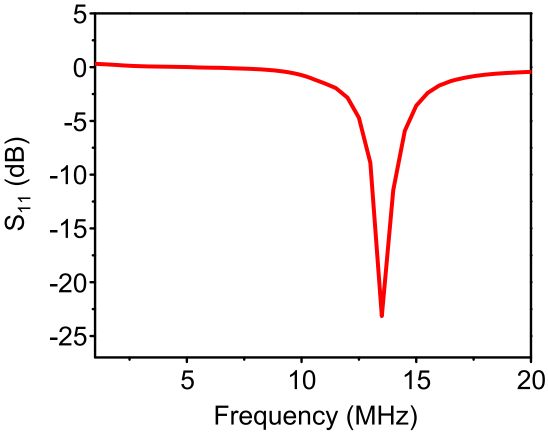

Supplementary Fig. 6 | S_{11} response with an Rx coil placed on the Tx coil shows a 23 dB return loss (matching) at a resonance frequency of 13.56 MHz, indicating an efficient wireless power transfer.

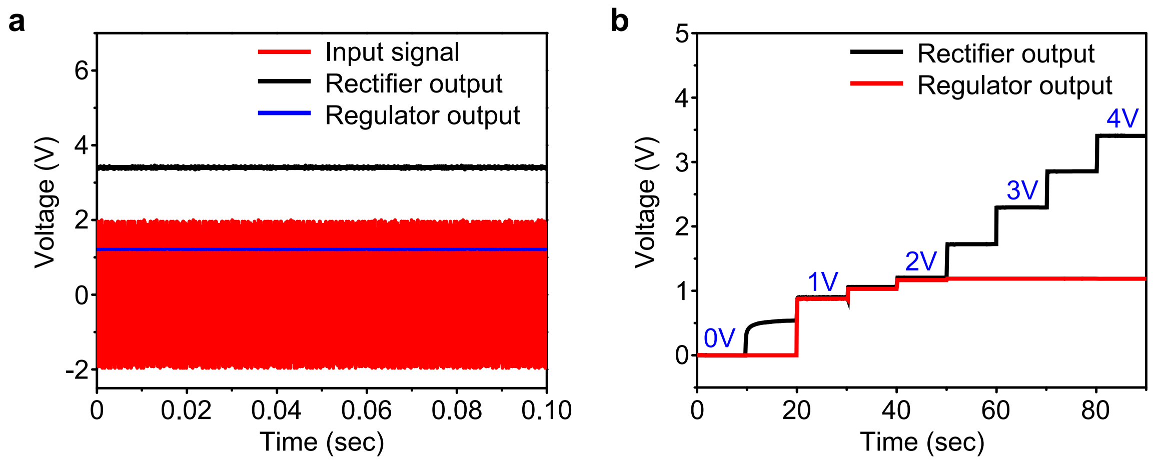

Supplementary Fig. 7 | a, Rectified signal output from wirelessly transferred AC signal with 4V. **b,** Rectified output (black) and regulated 1.2 V output (red) while different wirelessly transmitted peak-to-peak voltage (blue) is applied.

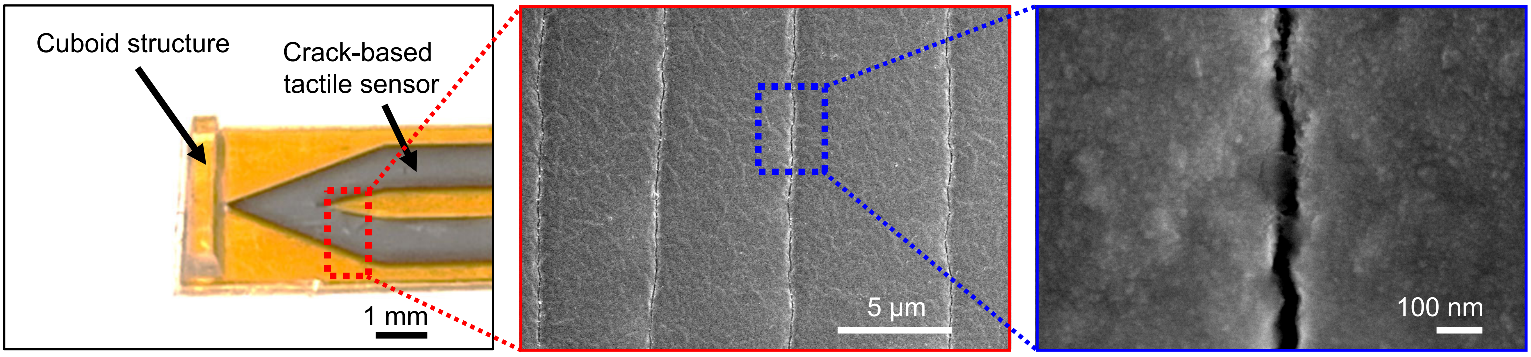

Supplementary Fig. 8 | Photograph of tactile sensor and SEM images of the cracks on the crack-based tactile sensor. When pressure is applied, the gap between the micro-crack widens, inducing the resistance change according to the intensity of applied pressure.

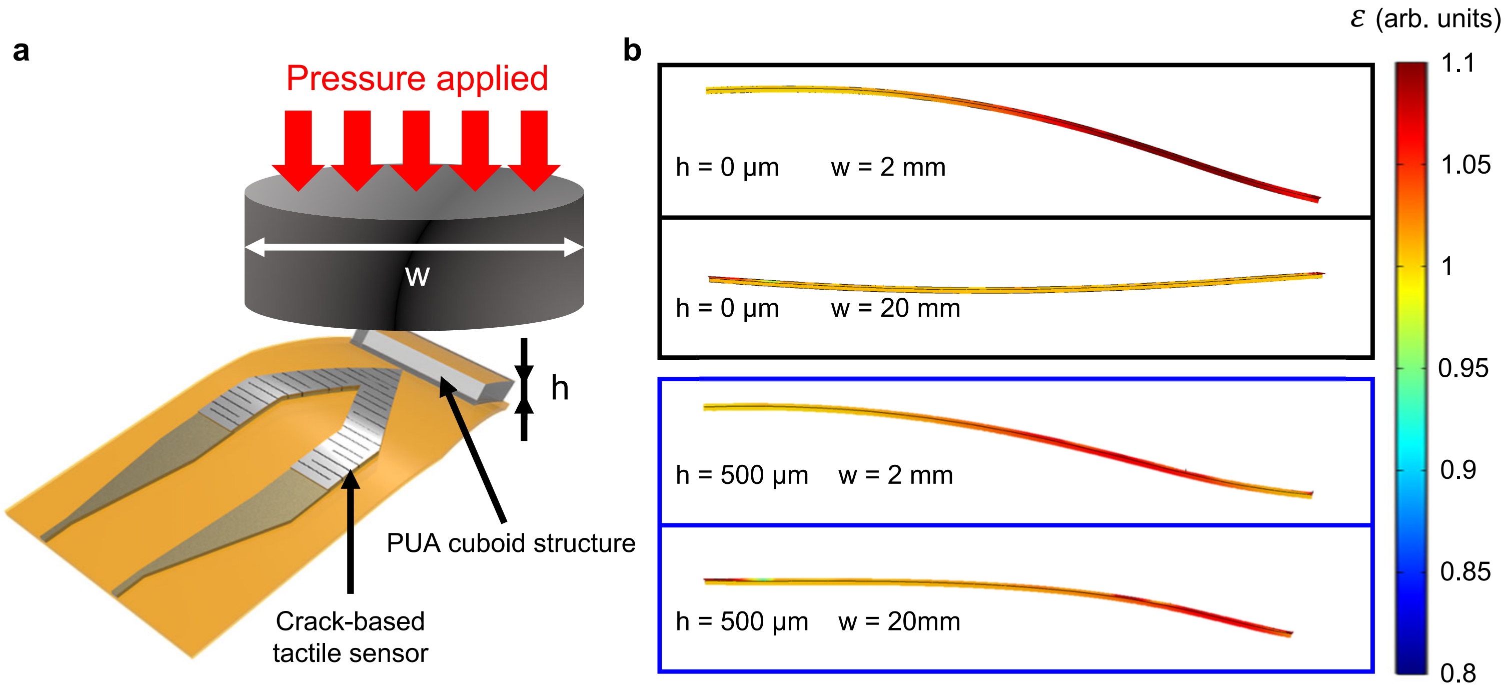

Supplementary Fig. 9 | a, Schematic illustration providing an overview of the experimental setup for the tactile sensor property test. w signifies the width of the rod, while h represents the height of the cuboid structure. **b,** FEM longitudinal strain results under different conditions. It compares sensors without and with a cuboid structure, denoted as h = 0 μm (black boxes) and h = 500 μm (blue boxes), respectively. The sensors were pressed by a rod with different diameters, namely w = 2 mm and w = 20 mm. In the absence of a cuboid structure, it can be confirmed that even when pressure is clearly applied over a wide area, such as w = 20 mm, no strain is formed on the sensor.

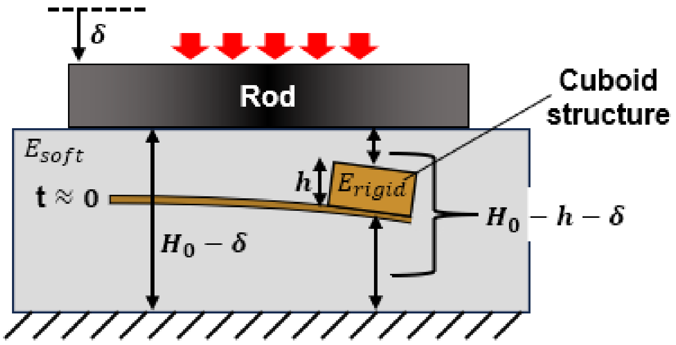

Supplementary Fig. 10 | A schematic image illustrating the theoretical operating mechanism of the cuboid structure. It displays images of the sensor's deformation with broadly applied pressure when a film-type sensor with a cuboid structure is embedded in soft material.

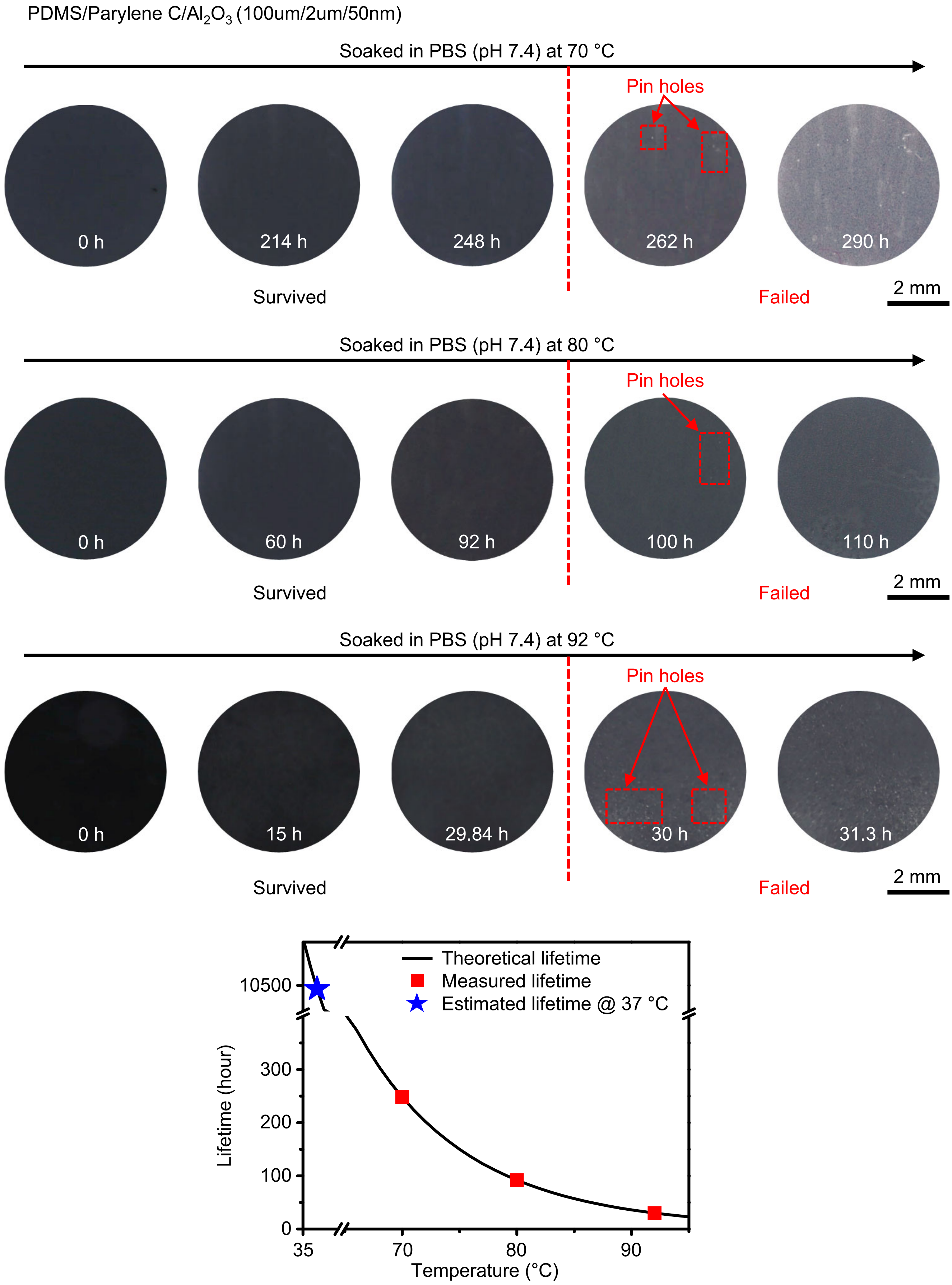

Supplementary Fig. 11 | PBS acceleration test for multilayer encapsulation. Mg, which instantly reacts with water, was used to determine the lifetime of the multilayer encapsulation. At 70 °C PBS, 80 °C PBS and 92 °C PBS lifetimes of the multilayer encapsulation layer are 248 h, 92 h, 29.84 h respectively.

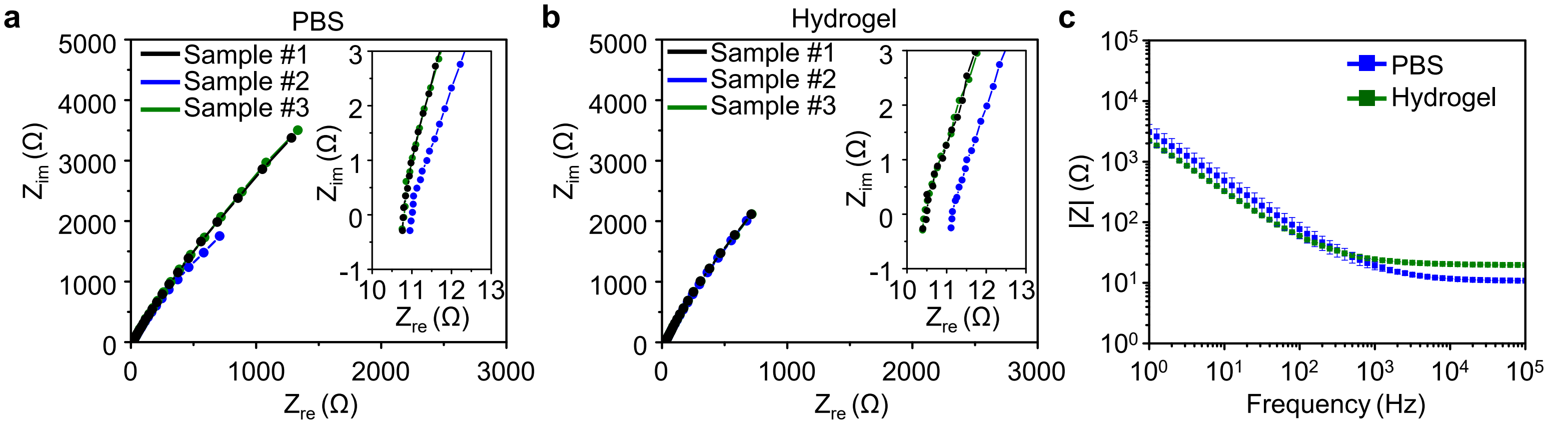

Supplementary Fig. 12 | Nyquist plots of the PBS (**a**) and hydrogel (**b**) (n=3). The range of frequency range was from 1 Hz to 100 kHz and inset graph shows same Nyquist plot data in the high-frequency range (from a few kHz to 100 kHz). (**c**) Bode plots of the PBS and hydrogel.

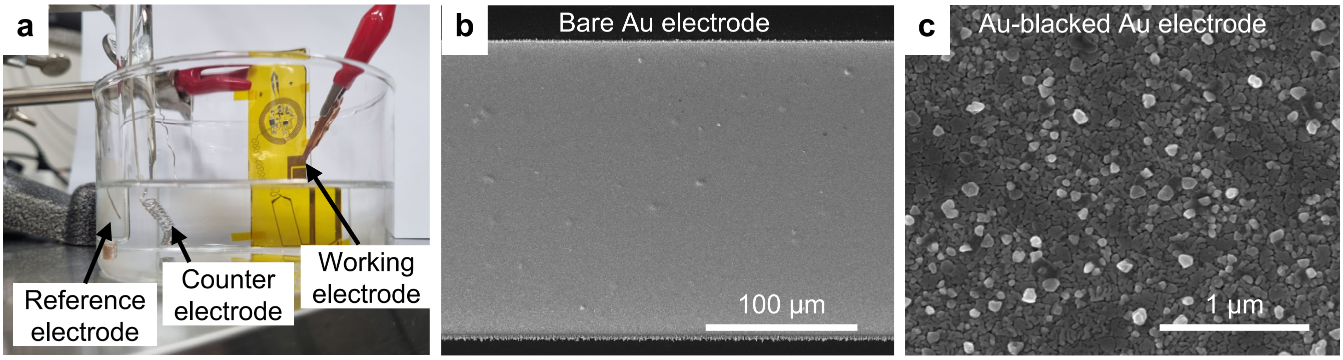

Supplementary Fig. 13 | a, Photograph of electroplating set-up. **b,c,** SEM images of the bare Au electrode (**b**) and the Au-blacked Au electrode (**c**).

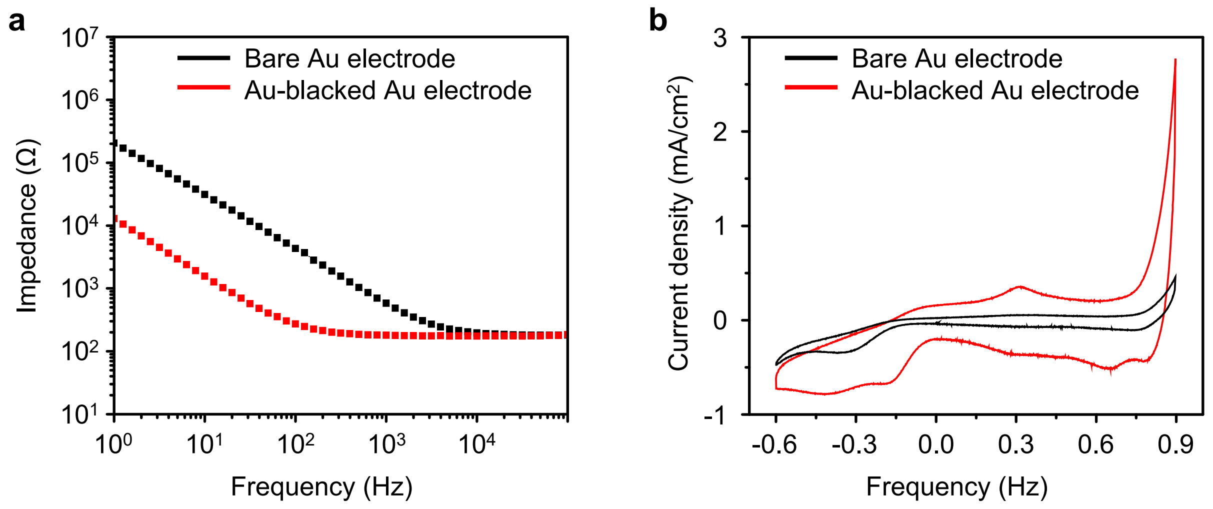

Supplementary Fig. 14 | a, EIS graph of the bare electrode and Au blacked Au electrode. Impedance of the Au blacked Au electrode decreases to 179.5 Ω at 1 kHz, which is ~31% lower than that of the bare Au electrode (578.8 Ω at 1 kHz). **b,** Cyclic voltammograms (C-V) of the bare electrode and Au blacked Au electrode (width: 0.26 mm, length: 7.5 mm).

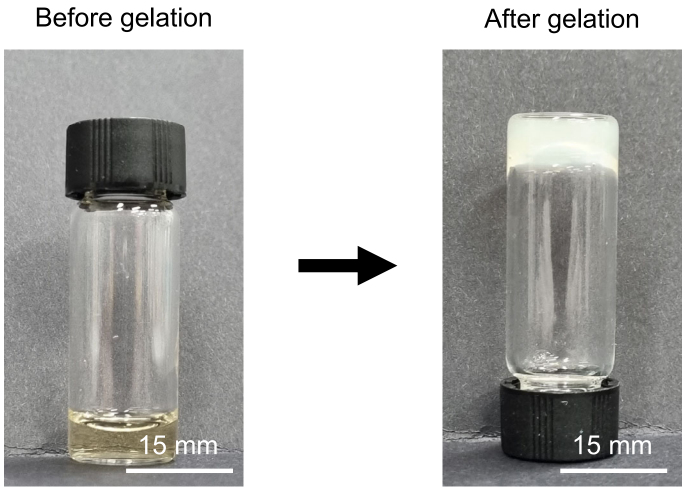

Supplementary Fig. 15 | Photograph showing the artificial skin before and after gelation. After gelation, collagen and fibrin are crosslinked together, which transform into a viscoelastic state.

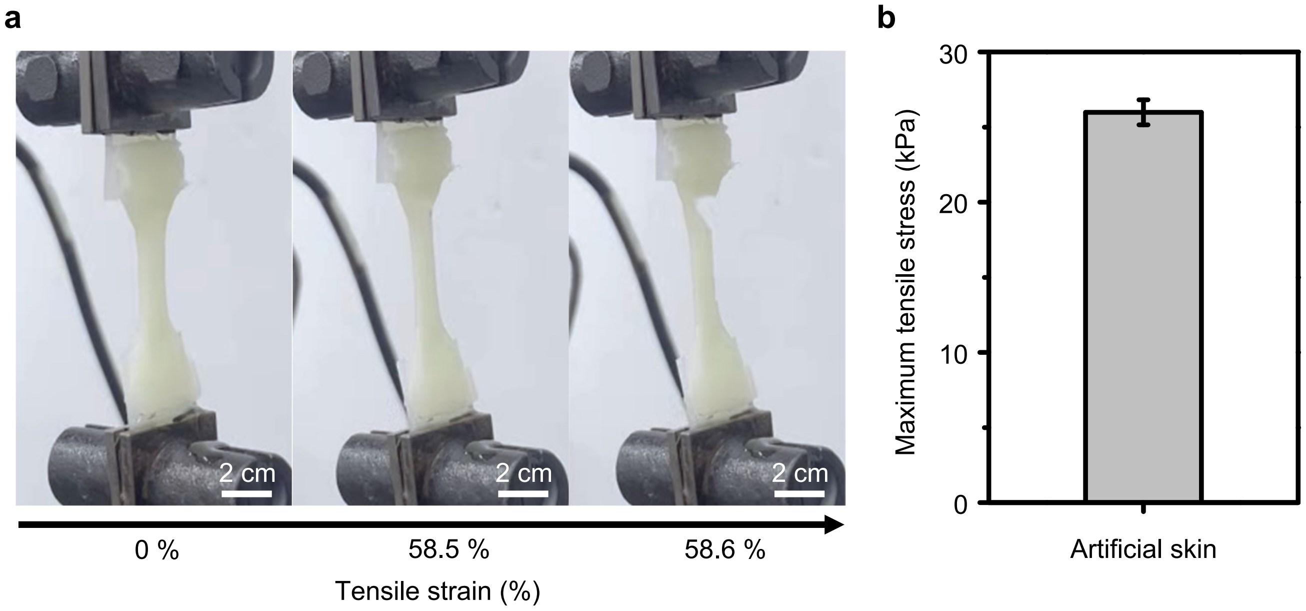

Supplementary Fig. 16 | a, Photograph of the tensile stretching test of the artificial skin. **b,** Average of the maximum tensile stress for the artificial skin (n=3)

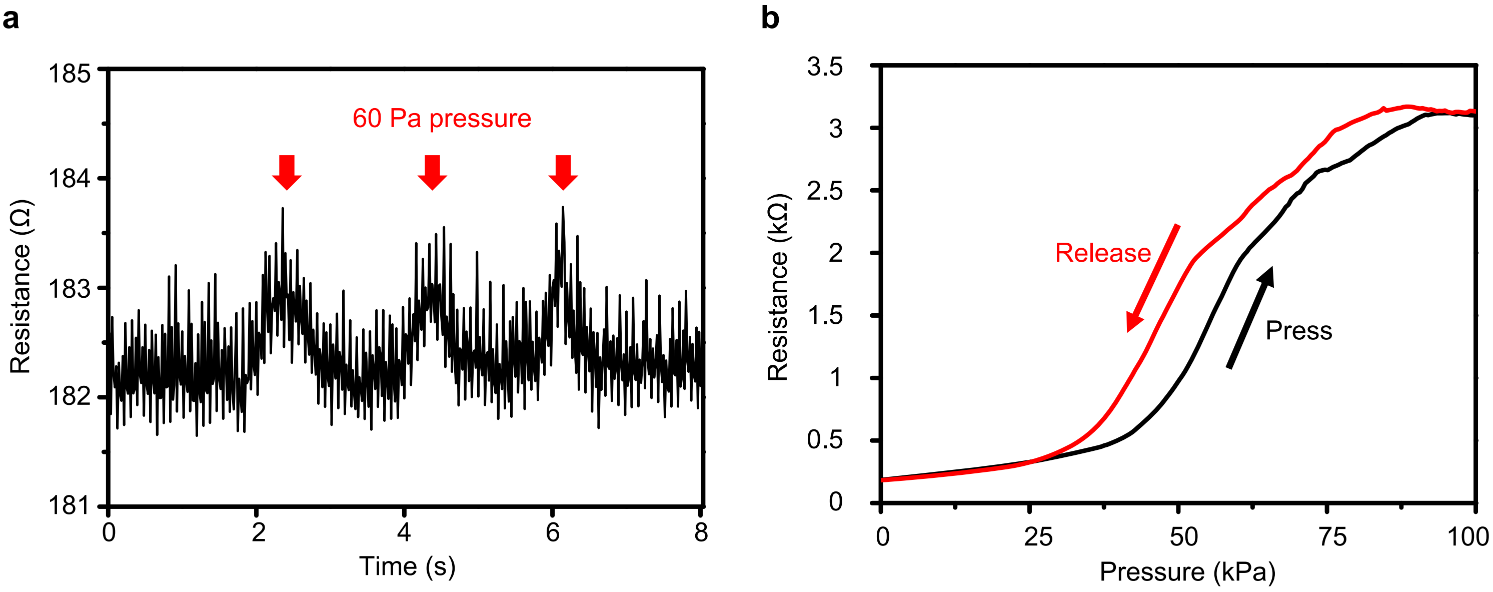

Supplementary Fig. 17 | Basic tactile sensor characteristics embedded in the CFAS. The minimum pressure detection is 60 Pa (**a**), and due to the viscous environment of CFAS, there is some level of hysteresis (**b**).

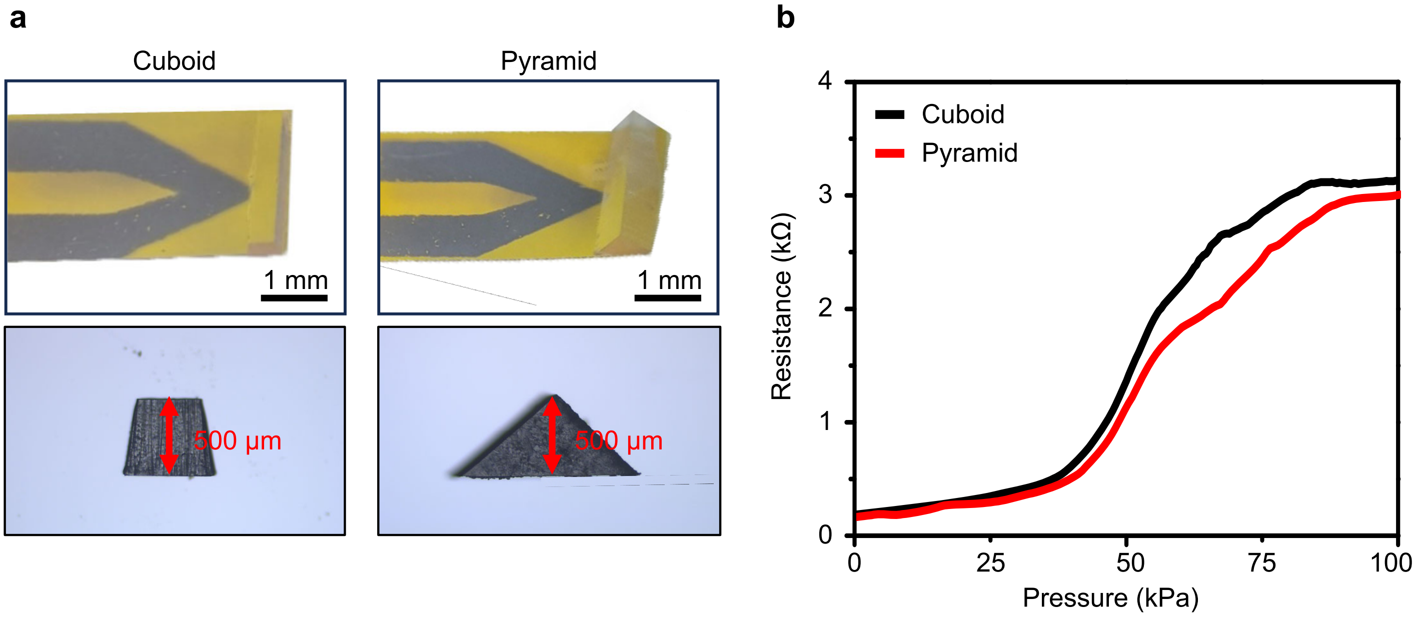

Supplementary Fig. 18 | The effects based on different structural forms. **a,** Tactile sensors with cuboid and pyramid structures. **b,** Resistance change graph embedded in CFAS.

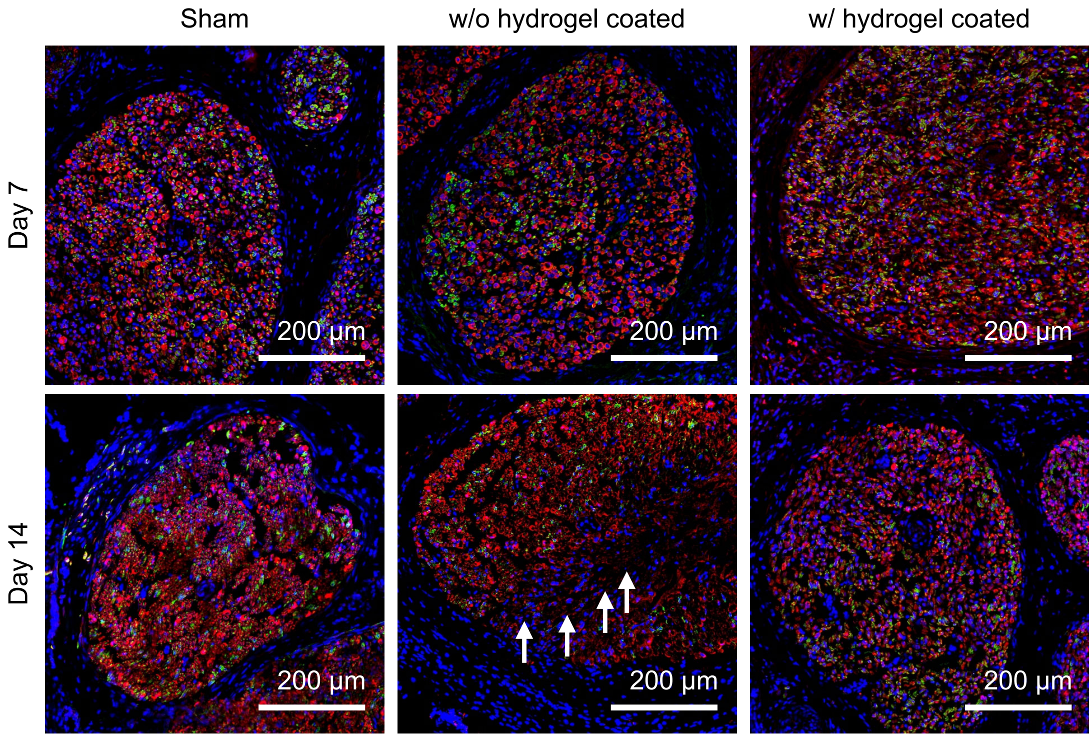

Supplementary Fig. 19 | Immunofluorescence staining of cross sectioned sciatic nerve in w/ and w/o hydrogel coated groups. (Red: S-100 (schwann cell), Green: β-tubulin (neuron), Blue: DAPI (nucleus), White arrow: degree of nerve damage)

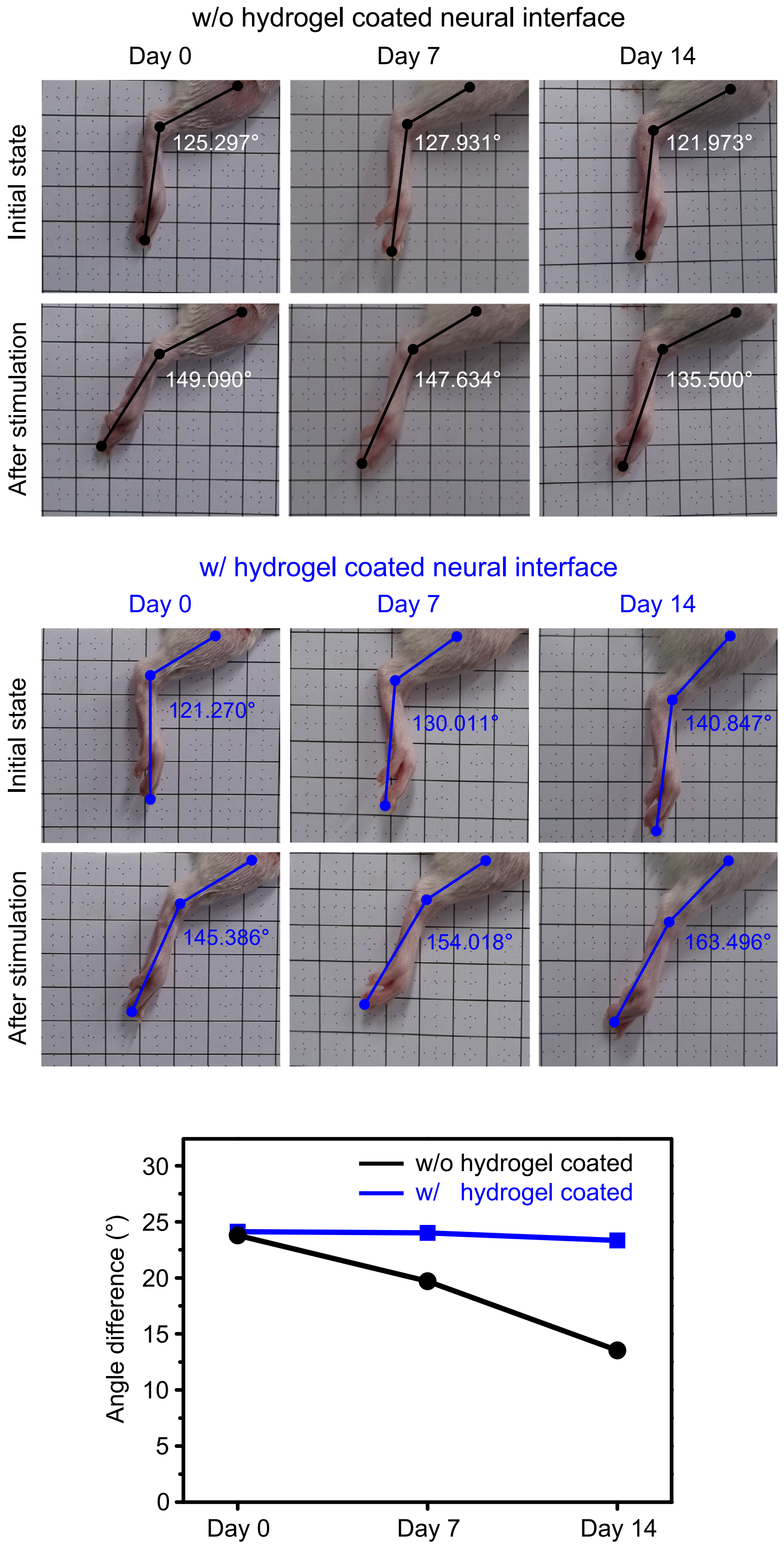

Supplementary Fig. 20 | Effectiveness of hydrogel coating on the neural interface for electrical stimulation after 14 days of implantation.

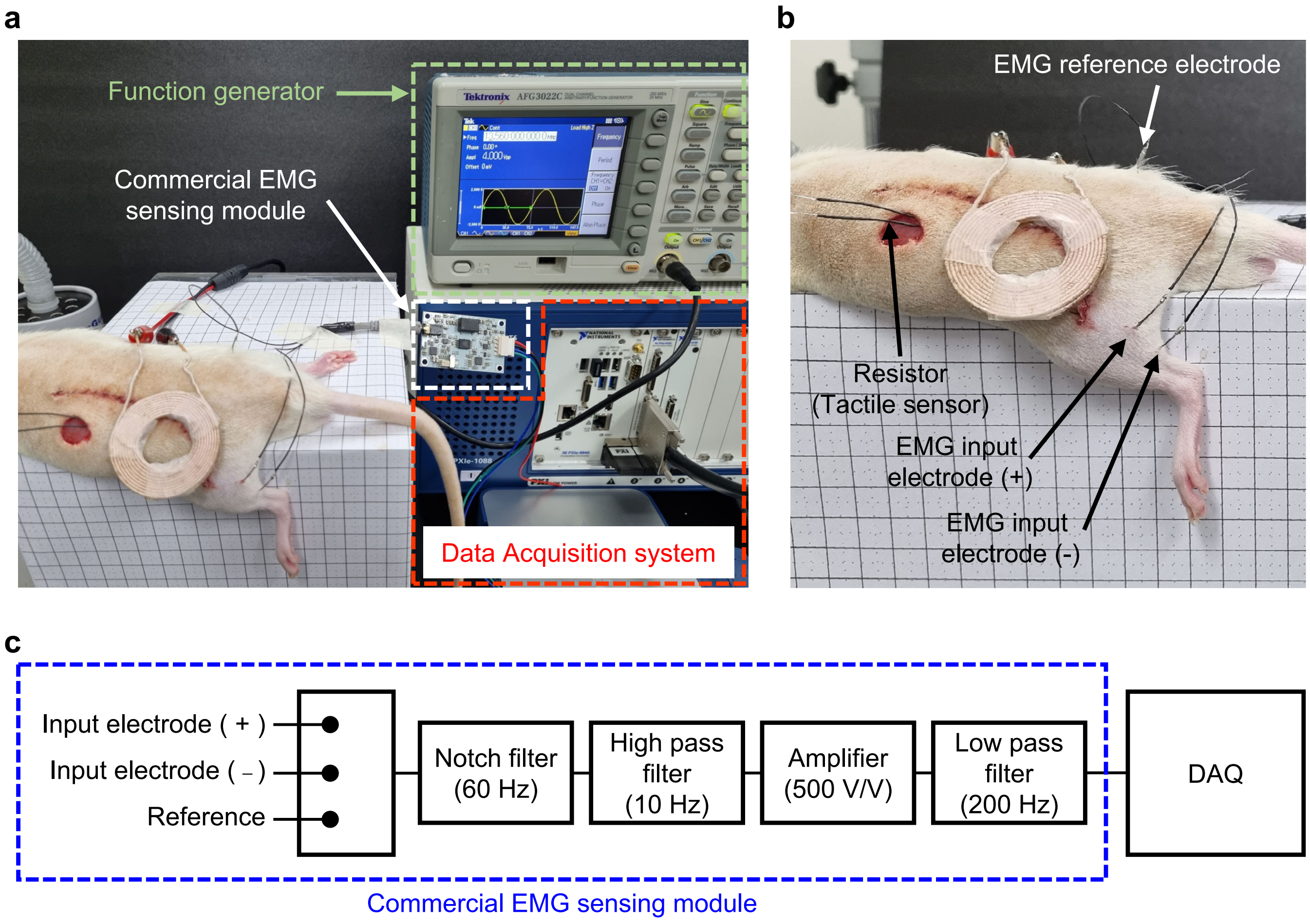

Supplementary Fig. 21 | a, Photograph of the EMG measurement setup. **b,** Magnified image of the EMG measurement setup. c, Block diagram of the commercial EMG sensing module.

Supplementary Note

Supplementary Note. 1 | The theory of the cuboid structure effect.

Considering the tactile sensor embedded in soft skin tissue or CFAS, it is possible to simplify this state as shown in Supplementary Fig. 10. E_{rigid} and E_{soft} are Young's modulus of the cuboid structure and the surrounding soft material, respectively. In our research, E_{rigid} is 52.19 MPa as PUA, while E_{soft} can be considered to be around 10 to 1,000 kPa for materials like skin, CFAS, or other soft biological tissues. When pressure with a flat and wide object is applied by the displacement of δ, the degree of compression varies depending on the presence of the cuboid structure. Here, we considered that the rigid cuboid structure has not compressed as Young's modulus of the cuboid structure is about 1,000 times higher than the CFAS in our experiment. Therefore, the compressed strain of the surroundings can be expressed as follows when we consider the sensor's thickness, t, to be negligible:

$$\varepsilon_{h=0} = \frac{\delta}{H_0} \tag{1}$$

$$\varepsilon_{h\neq0} = \frac{\delta}{H_0 - h} \tag{2}$$

H_0 is the thickness of the surroundings before pressure was applied, and h is the cuboid structure height. If we compare the two compressive strains, $\varepsilon_{h\neq0}$ is always higher than $\varepsilon_{h=0}$. It means surroundings where cuboid structure exists, get compressed more by higher concentrated stress as the surroundings are the same materials. In addition, this phenomenon becomes more pronounced as h increases due to the two comparison equations.

Supplementary Note. 2 | The theory of the encapsulation layer lifetime.

Calculating the lifetime of encapsulation is one of the important factors for implantable device. Since the body temperature of the animal is maintained at 37 °C, it is essential to assess the encapsulation lifetime at 37 °C. Therefore, we performed an accelerated PBS test using Mg which reacts immediately with water molecules. The lifetime of encapsulation layer is 248 hours, 92 hours and 29.84 hours at 70 °C, 80 °C and 92 °C respectively (Supplementary Fig. 11). The estimated lifetime of encapsulation layer can be calculated with the Arrhenius equation:

$$Lifetime = A \cdot e^{-\frac{E_a}{RT}} \tag{1}$$

A is constant, Ea is activation energy, R is gas constant and T is temperature. By substituting lifetime and temperature in equation (1), we get:

$$248 = A \cdot e^{-\frac{E_a}{8.314 \cdot 353.15}} \tag{2}$$

$$92 = A \cdot e^{-\frac{E_a}{8.314 \cdot 363.15}} \tag{3}$$

By calculating the equation (2) and (3), constant A is $1.533 \cdot 10^{-13}$ and activation energy (E_a) is 99909.629. We get the complete Arrhenius equation by substituting these parameters:

$$Lifetime(hour) = 1.533 \cdot 10^{-13} \cdot e^{-\frac{99909.629}{8.314 \cdot T}} \tag{4}$$

To confirm the linearity of calculated Arrhenius equation, estimated lifetime at 92 °C from equation (4) and measured lifetime at 92 °C were compared. The estimated lifetime at 92 °C is 30.07 hours while measured lifetime at 92 °C is 29.84 hours which showed only 0.7% difference indicating that calculated Arrhenius equation can estimate the lifetime of encapsulation precisely. Therefore, the lifetime of encapsulation layer at 37 °C is 10,296 hours from equation (4) which is 429 days.
