# Supplementary material for: Bionic artificial skin with a fully implantable wireless tactile sensory system for wound healing and restoring skin tactile function
Source: Nat Commun. 2024 Jan 2;15:10. doi: 10.1038/s41467-023-44064-7 (PMC10762199; doi:10.1038/s41467-023-44064-7)
Supplement: Supplementary file 3 — Description of Additional Supplementary Files [file 41467_2023_44064_MOESM3_ESM.pdf]

Description of Additional Supplementary files

Supplementary Movie 1 : Wireless operation of WTSA.

Supplementary Movie 2 : Leg movement difference for low and high intensity of applied pressure.

Supplementary Movie 3 : Resistance-pressure response from tactile sensor embedded artificial skin.

Supplementary Movie 4 : In-vivo demonstration of implanted WTSA
